# Supplementary figures and images for: Nucleus accumbens dopamine release reflects Bayesian inference during instrumental learning
Source: PLoS Comput Biol. 2025 Jul 2;21(7):e1013226. doi: 10.1371/journal.pcbi.1013226 (PMC12233953; doi:10.1371/journal.pcbi.1013226)

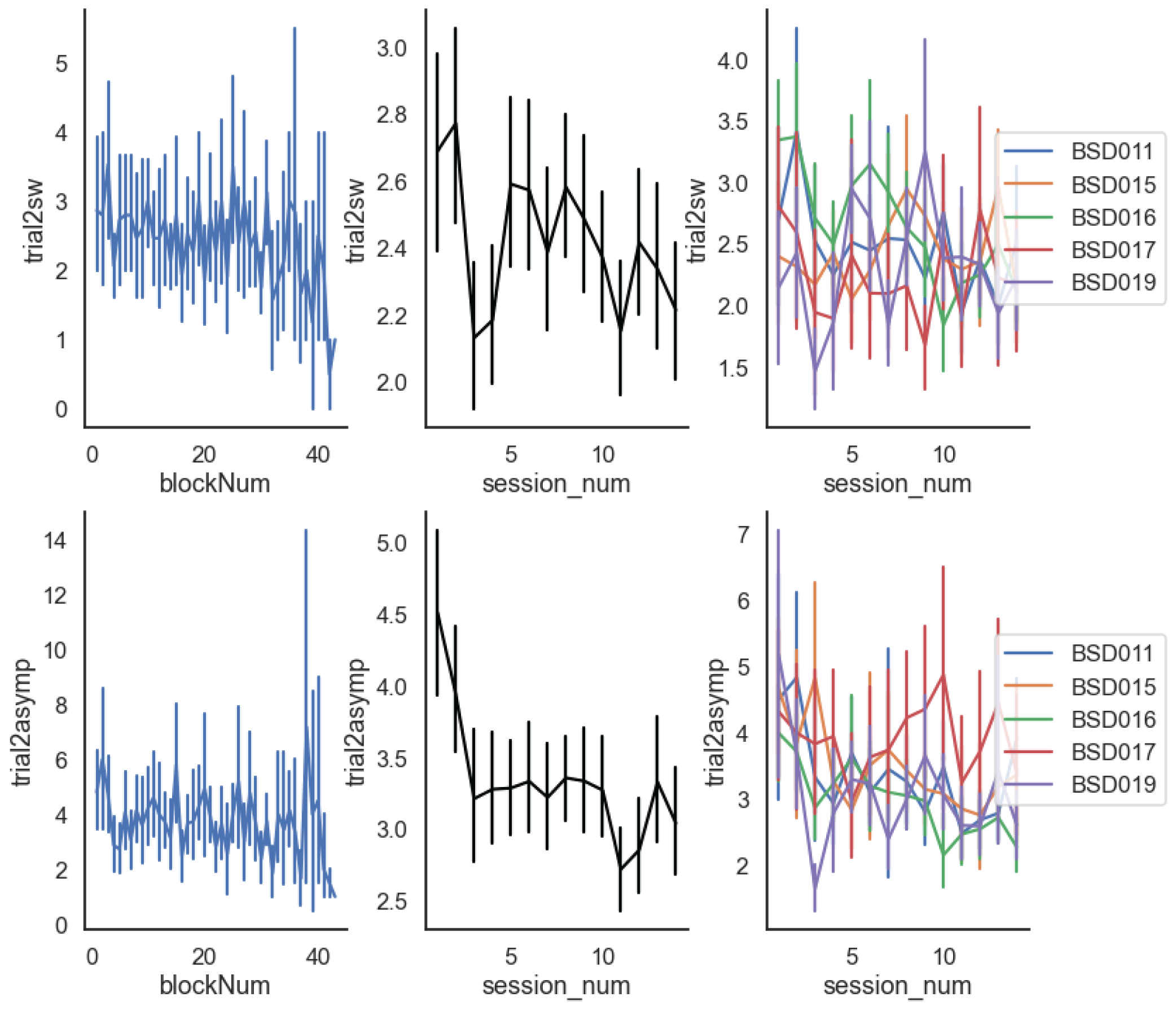

Supplement: S1 Fig — Top row y-axis shows the number of trials mice take to switch to the correct port in a new block (trial2sw). Bottom row y-axis shows a metric of the first trial from which the animal chose the correct port and then persisted with the choice selection for 2 subsequent trials consequently (trial2asymp). First column x-axis shows the relationship between the measures and the number of blocks within a training session, demonstrating within session learning. A downward trend for both measures suggests learning and faster switches across different reward blocks within session. Second column x-axis shows that the switch measures decreased as the number of training sessions increased, describing learning across multiple sessions. This is consistent with results in Fig 1F, where accuracy improved over multiple sessions. Third column x-axis shows the same thing as the second column, but separated by animals. Error bars show 95% bootstrapped confidence intervals. (PNG) [file pcbi.1013226.s001.png]

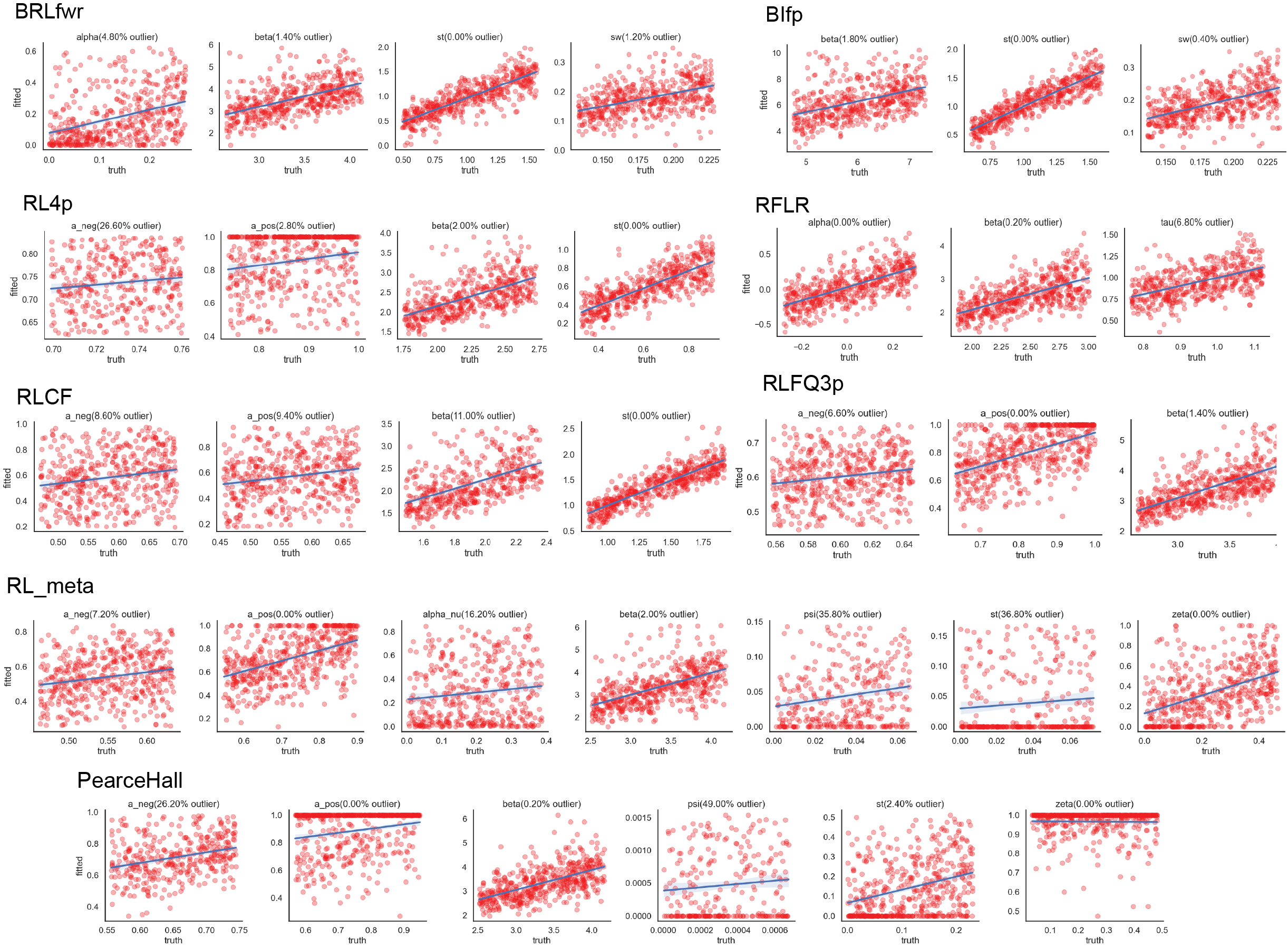

Supplement: S2 Fig — We took the maxima and minima for each of the best fitted parameter values for all subjects, and constructed a uniform distribution for each. We then sampled from these empirical distributions for simulating behaviors and fitted best fitted parameters for each set of simulated behaviors for each model. The results are generated after 500 runs of random parameter samples for each model, shown as a scatter plot with truth parameter against fitted parameters. Each row is a different model noted at the top left corner. Outliers, defined as 6 standard deviations from mean true parameter values, were thrown out for visualization purposes but percentages were noted. (PNG) [file pcbi.1013226.s002.png]

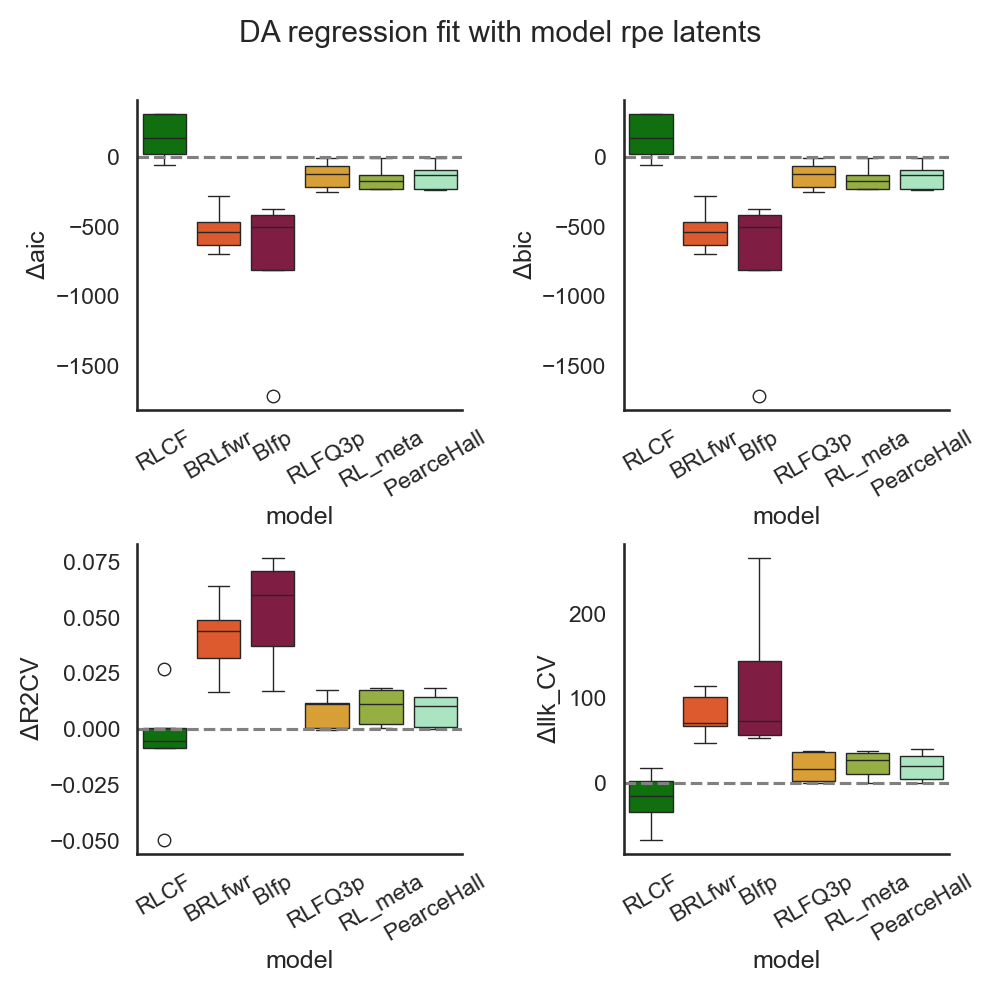

Supplement: S3 Fig — Similar to Fig 4F, we included results of model fitness using different metrics: relative AIC, relative BIC, relative cross validated R2 (R2CV), and relative cross validated log likelihood (llk_CV). All metrics converged on favoring Bayesian model predictions of dopamine at outcome phase. Error bars show 95% bootstrapped confidence intervals. (PNG) [file pcbi.1013226.s003.png]

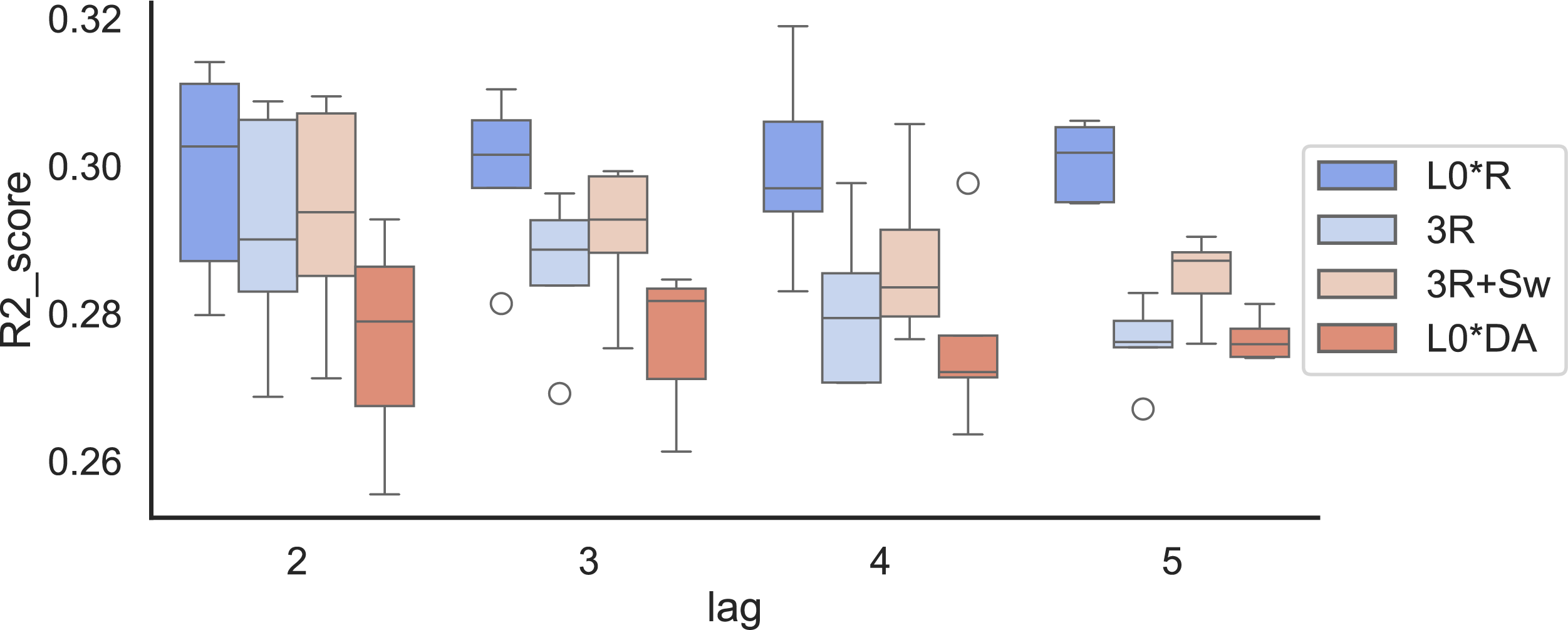

Supplement: S4 Fig — We compared the cross validated R2 score using different feature sets, while keeping the use of dopamine as output variable. L0:R: Standard formulation using N trial back choice and reward interactions. 3R: just R_chosen, R_unchosen, Reward features. 3R+Sw: in addition to 3R features, we included interactions of whether a trial is an animal switch trial, or animal stay trials. L0:DA: we used the interactions between past trial dopamine values and choice selections. Together we found that 3R+Sw was enough to capture similar levels of variance compared to L0:R. We used 4 lags because it allows us to capture a relatively high amount of past outcome levels, with only the expense of 1% variance explained. Error bars show 95% bootstrapped confidence intervals. (PNG) [file pcbi.1013226.s004.png]

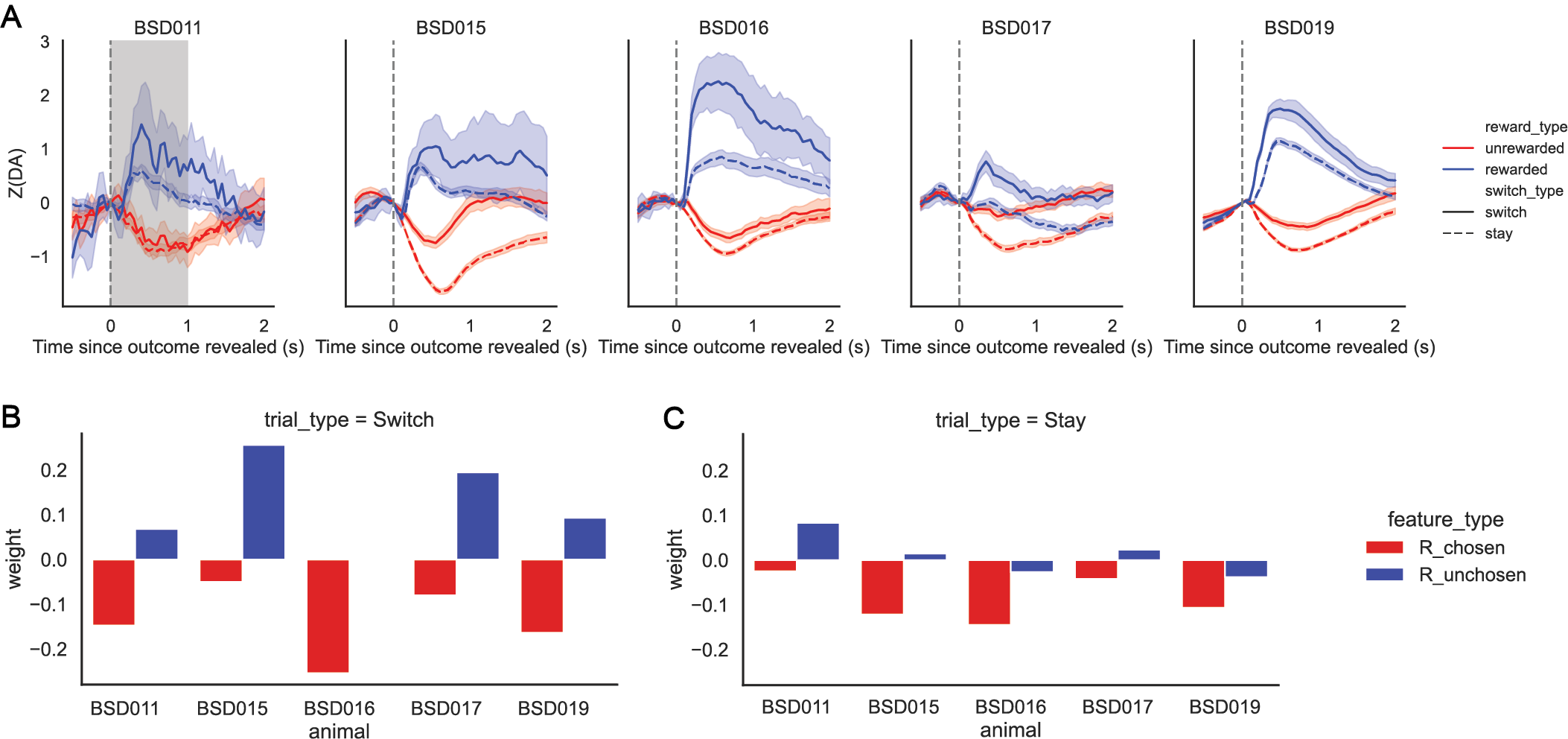

Supplement: S5 Fig — (A) Average differences in dopamine responses to rewards or unrewarded outcomes across switch stay trials are fairly consistent across animals. (B-C) Similar to LMER regression of influence of past rewards on dopamine responses, we did OLS for each animal separately. All but one animal showed a strong qualitative resemblance to Bayesian model predictions for Switch trials. As discussed in the main text, R_unchosen effect was highly variable across animals, and had a near-zero net effect, corresponding more to the predictions of BIfp simulation. Error bars show 95% bootstrapped confidence intervals. (PNG) [file pcbi.1013226.s005.png]

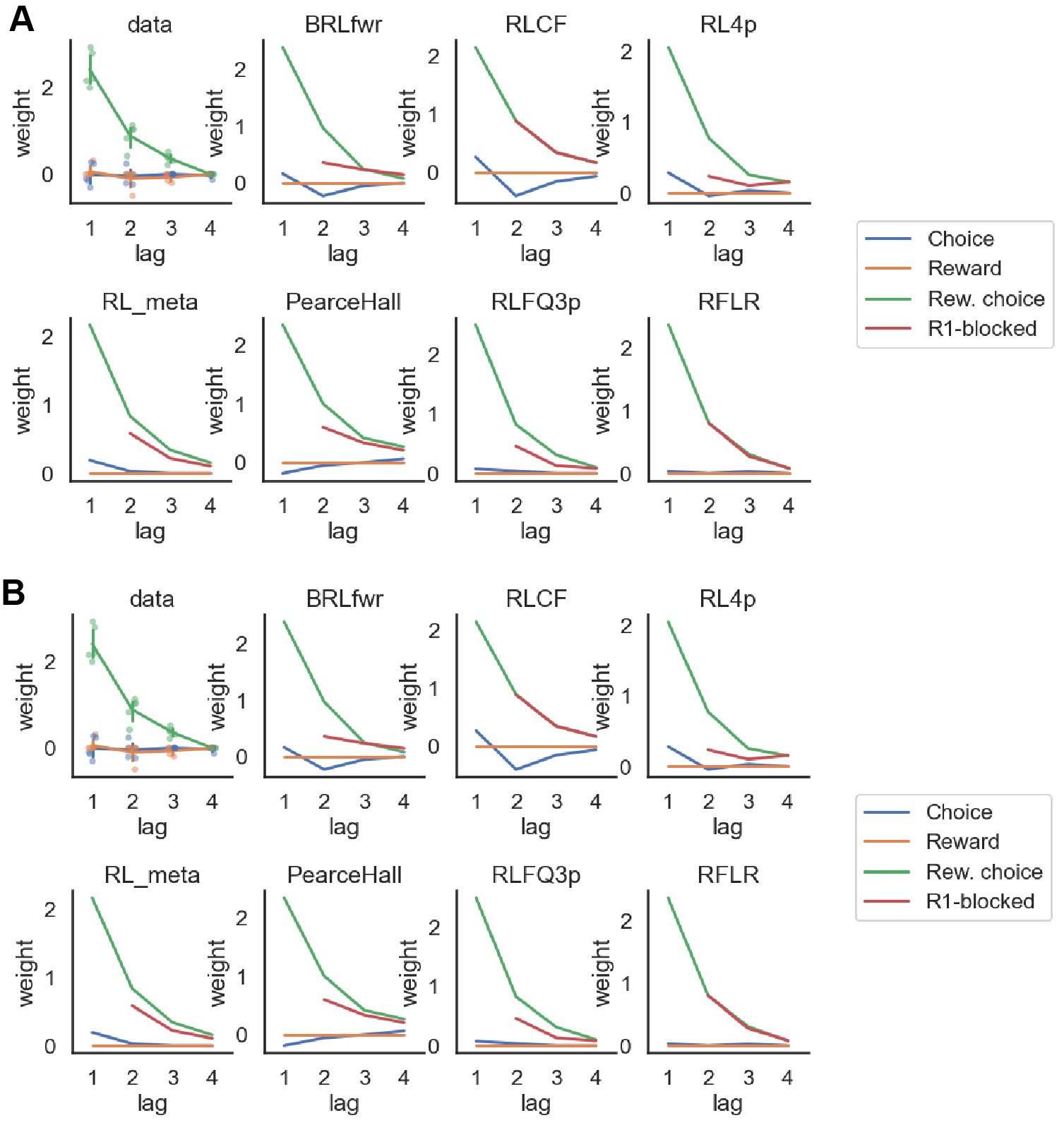

Supplement: S6 Fig — (A) Following the formulation from [19], we fitted logistic regression models to individual mice behavior data and identified consistent patterns of decaying choice outcome weights for more distant outcome histories. Error bars show 95% bootstrapped confidence intervals. Specifically, we used this formula (C~t−i=2Ct−i−1): Ct=∑iβiCRC~t−iRt−i+βt−iRRt−i+βt−iC~C~t−i. (B) To identify the blocking effect of the reward at trial t-1 found in [32], we fitted the following regression model, where βiCR+ represent the R1-blocked coefficient: Ct=∑i=2pβiCR+C~t−iRt−i1[Rt−1=0]+βiCR−C~t−iRt−i1[Rt−1=1]+∑i=1pβt−iRRt−i+βt−iCC~t−i (PNG) [file pcbi.1013226.s006.png]

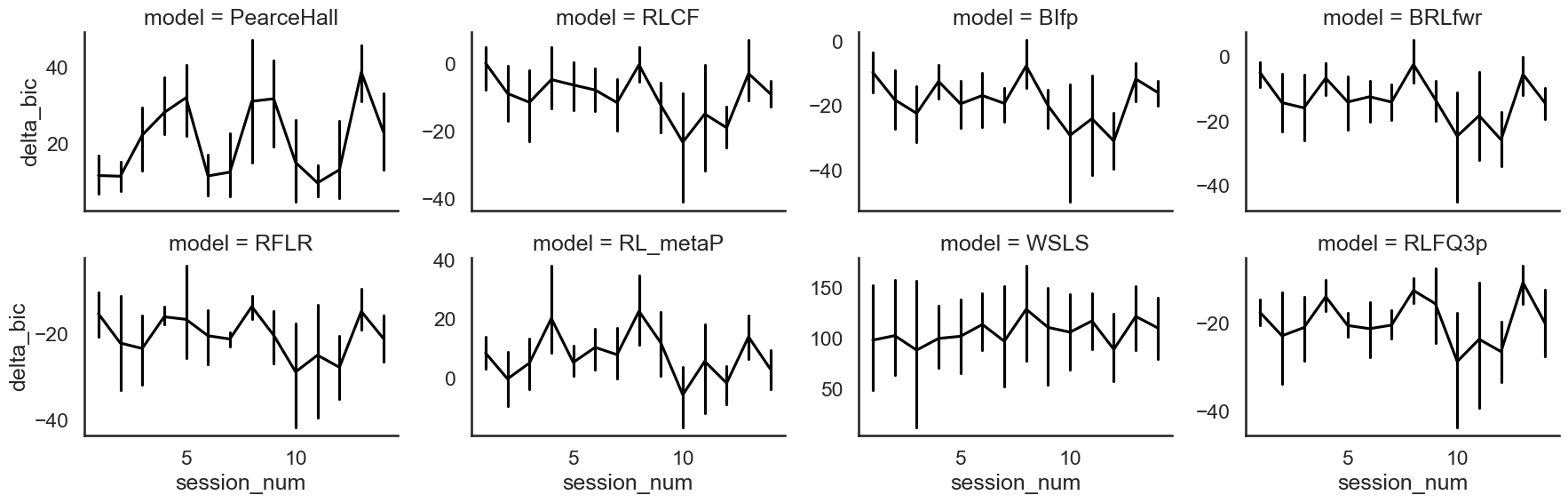

Supplement: S7 Fig — To test if there was meta-learning across sessions, we fitted cognitive models to mouse behavior across multiple sessions. We did not find evidence of any significant and consistent increase in model fitness across multiple sessions. We speculate that mice were able to learn the inference structure of the task during a brief pre-training phase, when the reward probability of the high value option changed from 90%, 80%, to 78% (Fig 1B). Model fitness of Bayesian models did show some decline in some later sessions but then recovered, suggesting that there was no sustained change in behavioral strategy after extended training. Error bars show 95% bootstrapped confidence intervals. (PNG) [file pcbi.1013226.s007.png]

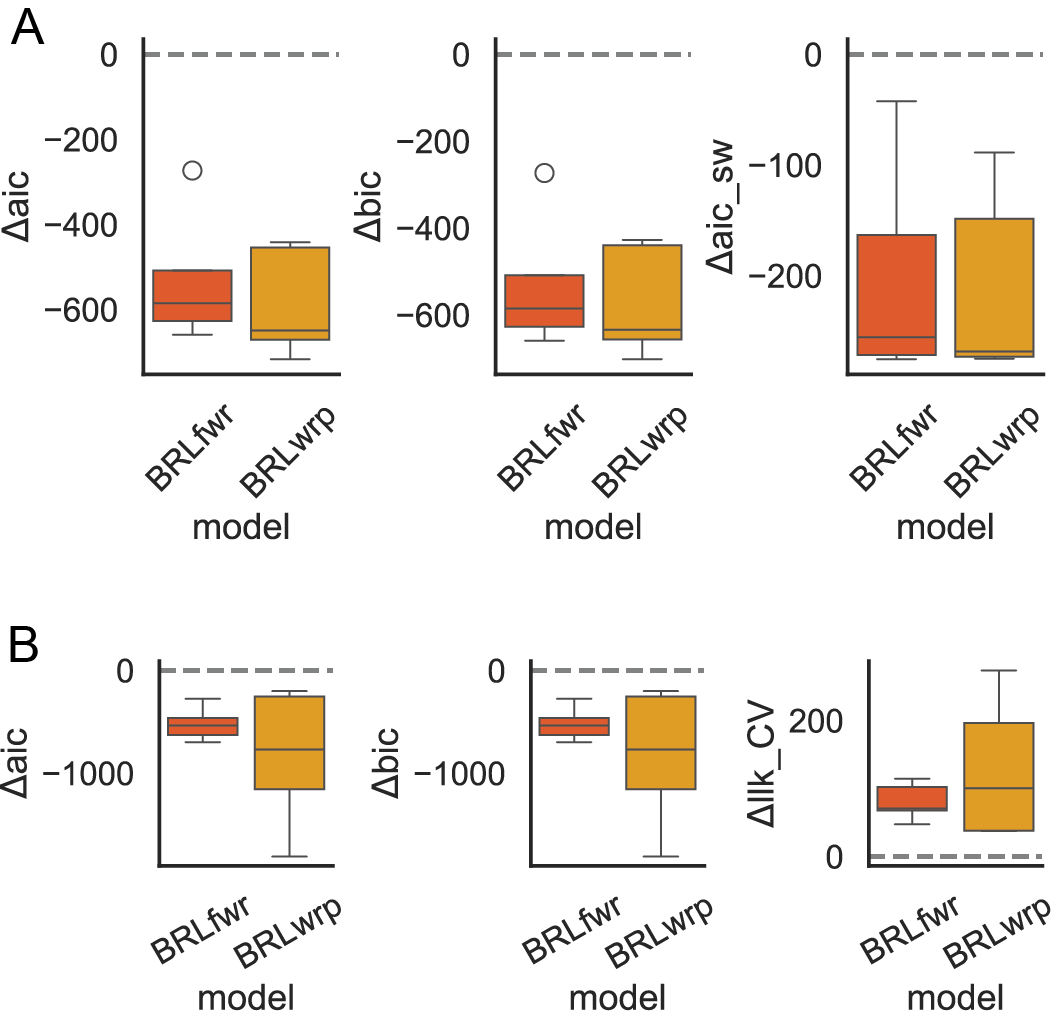

Supplement: S8 Fig — To further justify that the success of Bayesian models (e.g., BRLfwr) is not solely due to setting the initial reward probability of high value state as ground truth, we fitted BRLwrp, where both rewarded weights as well as reward probabilities are fitted to data. No statistical differences were observed between BRLfwr and BRLwrp in their ability to explain mice behavior and dopamine data. Error bars show 95% bootstrapped confidence intervals. (PNG) [file pcbi.1013226.s008.png]
