# Supplementary material for: Nucleus accumbens dopamine release reflects Bayesian inference during instrumental learning
Source: PLoS Comput Biol. 2025 Jul 2;21(7):e1013226. doi: 10.1371/journal.pcbi.1013226 (PMC12233953; doi:10.1371/journal.pcbi.1013226)
Supplement: Attachment — Submitted filename: QU_belief_state_rebuttal.pdf [file pcbi.1013226.s009.pdf]

Reviewer #1

The authors investigate mice's behavioral strategies during a reversal task, specifically examining whether the rats use reinforcement learning (updating the value of each state) or Bayesian inference (inferring the hidden state). To determine this, they fit observed rat behavior with multiple models and compare the goodness of fitting. In addition, dopamine signals from the NAcc were recorded as a neural measurement of RPE, and then compared to the (pseudo-) RPEs generated by the models. The results indicate that the BLfp and BRLfwr models outperform other models, suggesting that Bayesian inference plays a crucial role in explaining rat behavior. The paper is generally well-written and easy to follow, but there is a concern that the assumptions underlying the reinforcement learning models do not capture the task's structure, which makes them appear as straw man, diminishing the significance of this study.

We thank the reviewer for their thoughtful comment on our paper being "well-written" and "easy to follow".

Overall, the models which we collected to compared to Bayesian models were chosen not as a strawman/strawmen, but rather because they contain a rich set of cognitive mechanisms and have been shown to perform well in explaining human and mouse behavior in 2ABT tasks similar to our own (Eckstein et al. 2022, Beron et al. 2022). These models included forgetting mechanisms (RFLR, RLFQ3p), asymmetrical learning rate (RL4p, RLFQ3p, RLCF, RLmeta, PearceHall), dynamic learning mechanisms (RLmeta, PearceHall), and importantly counterfactual RL (RLCF, which can be viewed as an approximation for Bayesian learning). Given these benchmark models have been quite effective at explaining behavior we would argue that their comparison with BRL/BI models is a fair comparison.

In the task, the reward probabilities are 75% and 0% for two options, respectively. At least two critical aspects of the task structure are implicitly incorporated into the Bayesian models but not represented in most RL models. The first one is that the reward probabilities for the two options are anti-correlated. A higher reward probability on one side directly implies a lower reward probability on the other side. While the RLCF model takes this into account, most other RL models do not. Second, receiving a reward on a single trial from one side implies that this side is rewarded while the other side is non-rewarded. The Bayesian models also have access to the exact reward probabilities, which provides further precision in their predictions.

Based on these reasons, it appears that the better fit of the Bayesian models in this study primarily suggests that the rats learn the underlying task structure rather than using a Bayesian approach to estimate the hidden state or perform pure Bayesian inference.

To enable a fairer comparison, it would be valuable to include RL models that incorporate the critical aspects of the task structure, such as the BRL models mentioned in the manuscript, but estimate the hidden states in a non-Bayesian manner. Such models would help determine whether the observed behavior is better captured by Bayesian inference or by structural learning.

We thank the reviewers for these thoughtful comments and appreciate the chance to respond. We think that the RLCF model may achieve the goal set by the reviewer. The RLCF model incorporated a counterfactual mechanism, which allowed the subjects to treat negative outcomes in one point as a simultaneous positive outcome at the alternative choice. This is an alternate way for an RL family model to estimate the task structure in a non-Bayesian manner (as noted previously by Eckstein et al., 2022). Nonetheless, BRL was able to explain the mouse behavior and neural data better than RLCF (Fig 2C, Fig. 4F).

We would also like to point out that, though the BRL model has access to the exact probabilities, that does not automatically make them the best fitting models, since the mice may not necessarily follow the optimal strategies in their behaviors. For instance, all models were able to explain the mice behaviors better with choice stickiness, which is more biological than decision-optimal. Furthermore, we also fit the BRL models where they do not have access to the exact probabilities, but achieved similar model fitness (Fig. S8)

Overall, our model comparison does not aim to generate a “super” model with all mechanisms mentioned above, but, following the principle of Occam’s razor, merely to pinpoint which mechanisms, when used alone in a model, can explain the most amount of variance of mouse behaviors. Despite the fact that our BRL model does not have asymmetrical learning rates or dynamic learning, it is able to capture more variance than any other learning mechanisms mentioned above. We think this observation will be of value to the journal readers.

Other concerns:

1. It would be interesting to model the initial learning process. Rats may form a representation of the left-rewarded and right-rewarded states after repeated reversals, but likely not before the first reversal. Additionally, the exact reward probability is unknown to animals when they are making inferences, and it should be something they learn over time. If the authors could demonstrate the transition process from a RL approach to an RL-Bayesian inference hybrid or a pure Bayesian inference model, and illustrate how dopamine activity changes during this transition, it would greatly enhance the significance of this study.

We thank the reviewers for the helpful suggestion. In response, we analyzed the model fitness across sessions. Although we did start dLight recording very early in training, we found no significant improvement in relative BIC over sessions (Fig. S7). Instead, Bayesian models consistently explained mice behavior data better than the RL4p benchmark. Although we did start recording dopamine data very early in training compared to most studies of bandit tasks, we, in hindsight, regret not starting even earlier because it is possible that mice learned the block switch structure very quickly during what we call “pretraining”. We would argue that it is still of interest to know that mice can form these representations after only ~10 days, and that this may suggest an advance in understanding mouse cognition with utility for translation. We modified the discussion section to reflect these issues:

“Moreover, our experimental design incorporated a multi-stage pre-training protocol before we started recording dopamine data in the "Full task" condition. Mice started with 100% reward probability, and it was brought down in stages to 90%, 80% and then 78%, before the “full task” at 75%. Since this pretraining took usually approximately 10 days on average, this experience was possibly enough for mice to develop Bayesian inference strategies before dopamine recordings started. As a result, our current dataset cannot address the question of whether in the earliest days of pretraining, mice transitioned from initial reinforcement learning to a more sophisticated belief-state inference approach (Supp. Fig. S7).”

2. The authors include a meta-learning model to modulate the learning rate for negative RPE. However, why not modulate the learning rate for positive RPE as well? Additionally, models such as Mackintosh's and Pearce-Hall have been successful in explaining differences in learning rates across various tasks. Including these models could provide valuable insights and strengthen the conclusions of the study. Or at least explain why not include them.

We thank the reviewer for this comment and we recognize the importance of models that modulate learning rates for both positive and negative RPEs. We now include the Pearce-Hall model in the main figures to support the claim that BRL outperforms Pearce-Hall as well, which includes dynamic learning for both positive and negative learning rates.

3. Line 284: how is peak of dopamine transients calculated? Please also briefly mention it in the main text.

We apologize if this was not clear. We described the dopamine transient peak trough method in the main text results: “To simplify the testing procedure, we obtained dopamine summary statistics by taking the peak (as the maximum value) of dopamine transients for rewarded trials and troughs (as the minimum value) for unrewarded trials (dubbed DA-PT) during the one second window after the outcome.”

We are happy to further clarify if this is still too unclear.

Reviewer #2

The goal of this research is to determine whether dopamine concentrations in the Nucleus Accumbens core reflect reward prediction errors conditioned on the belief of the hidden state of the external world. The authors proceed to answer this question by training mice in a two-armed bandit task, modeling animals’ behavior using different types of models, and performing fiber photometry recordings using a dopamine sensor in the Nucleus Accumbens core. While the

results point towards the conclusion that inference-based methods explain both behavior and dopamine concentrations, as shown by previous literature, the evidence in this manuscript is a bit weak, and this research alone does not provide a conclusive answer.

We thank the reviewer for their time and consideration of the manuscript. We argue that it is of strong interest to the community regarding when and where the brain may use Bayesian inference to solve decision making tasks. Similar claims have been made recently (most notable this past year Blanco-pozo et al. 2024, Mishchanchuk et al. 2024) and these have been accepted in high impact journals using similar lines of argument to our current manuscript.

We think our manuscript adds to this exciting literature through 1) replication of results in a different task and 2) through a comprehensive comparison of a breadth of models featuring distinct learning mechanisms within the main manuscript (See also Reviewer SupplementaryTable 1 below).

1. As we understand, replication and thorough computational analysis is valued by PLOS. Also, use of state inference may not necessarily be expected to be used in all tasks, especially when the task is a simple two armed bandit task with rapid trial structures. Also changes in manipulanda such as lever press versus nose poke can create unforeseen differences. Therefore, it is of note that even in our bandit task which is simpler and faster than the task in Blanco-pozo et al. 2024 and Mishchanchuk et al. 2024, our models suggest mice appear to use Bayesian inference to solve it. Thus, our current manuscript adds new robustness to the conclusion that Bayesian inference is as a mechanism to explain mice behavior and dopamine (and hence value estimation in the brain).
2. We also assume the readers of PLOS will appreciate comparison of a wide array of cognitive models. In past work these different models are often evaluated in a somewhat isolated manner, but our work fits well in the scope of the PLOS Computational biology journal. We used this opportunity to perform a broad comparison to provide a consistent account across both neural and behavioral data, which together offers stronger arguments to support this emerging view of mouse cognition.

The topic of whether mice can perform Bayesian inference (or an approximation of it) and if dopaminergic RPEs are conditioned on it, as opposed to the traditional model-free computations, is of interest to the community, as shown by the growing literature pointing towards that direction. The authors state that the limitations of previous research on the topic are the lack of accounting for Bayesian inference models in 2ABT and comparing only a few models. However, the authors fail to acknowledge Blanco-Pozo et al. 2024 - https://doi.org/10.1038/s41593-023-01542-x, where they tested different RL variants and also Bayesian inference models, performed fiber photometry recordings on the dopamine system,

and concluded that both mice behavior and dopaminergic RPEs are consistent with a Bayesian inference account.

We thank the reviewer for the comment and firstly note that we did cite Blanco-Pozo et al. 2024 as prior relevant work in our first submission. As we noted in response to comments above, our work provides additional evidence for Bayesian inference in a simpler (non two step) task. Despite the simplicity of the task in our work, behavioral and neural evidence still converge on a Bayesian explanation, which further stresses the plausibility of the use of Bayesian processes in the mouse brain. Additionally, Blanco-Pozo et al. 2024 focused on comparing Bayesian models against model-free and model-based strategies, whereas our paper explores a wider array of RL models featuring distinct learning mechanisms that have shown to be effective at explaining mouse behaviors. To highlight the differential contribution and recognition, we added the following sentence to the end of introduction.

"Recently, Blanco-pozo et al. compared Bayesian models against model-free, model-based, as well as hybrid RL models in a two-step task. **To further elucidate how specific mechanisms, like asymmetric learning rate, counterfactual learning, dynamic learning rate, forgetting, and Bayesian inference, each differentially contribute to explaining mouse behavior and dopamine release dynamics in the nucleus accumbens in a simpler operant task (the solution of which does not specifically demand Bayesian inference**), we included a broad set of complex RL models commonly used in cognitive modeling."

Reviewer Supplementary Table 1:

Recent papers	Models compared	Task	Some key differences from our current manuscript
Mishchanchuk et al. 2024<br>Mouse (some of us are co authors)	RL4p, RLCF, RLFQ, Pearce-Hall, State-inference (BI)	2ABT<br>Lever press<br>Long trials	Our task is on a faster time scale, uses different manipulanda, focuses on model comparison in main text
Blanco-pozo et al. 2024<br>mouse	MF (~Q learning), MB, hybrid, bayesian model	2-step task	Our task is simpler and compares a wider array of models with distinct learning mechanisms.
Beron et al. 2022<br>Mouse	RFLR, RL4p, HMM, RLFQ	2ABT<br>Mouse<br>Nose poke	Task is similar to ours. Our manuscript explores further models and examines dopamine release as well as behavior.
Eckstein et al. 2022 (some of us are co author)	RLCF, BI	2ABT<br>Humans<br>Game	Our manuscript is in mouse, Eckstein et al. is in human; yet the task and the best fit model

		controller	matches, adding valuable translational connection. Our study also adds the measures of dopamine release.

While Qü et al. description of different RL and Bayesian inference models, how similar and different they are, can be of importance for the community, the evidence provided in this manuscript is not completely convincing. Despite authors using a wide range of models, they are not able to differentiate between models. As stated in line 144 - behaviorally, the 2ABT used in this research cannot discriminate between models, just that any other model is better than the traditional model-free RL. While using the dopamine signals to arbitrate between models points towards inference models to better explain the signals, not all analysis supports a particular model. So it is not clear what is the advantage of using all these models if one cannot arbitrate between them. The low N in this research and, consequently, the low statistical power, also limits the results.

Thank you for the comment. We would respectfully argue that a major goal is still met. Even though behavior analysis was not able to completely dissociate all the models, neural and behavior analysis yielded consistent evidence favoring Bayesian models against the alternatives.

Additionally, we can argue that our study does have limits in terms of total mice but we do have a large number of trials and repeated days of recording. Convergent evidence in neural and behaviors in the same animals, as well as consistency across animals (Fig. S5)**,** reinforces the claims of this paper. *Moreover, this paper is meant to add to the rich literature supporting the brain's use of Bayesian inference as a learning mechanism with a comprehensive model comparison, rather than to simply provide conclusive results on its own.*

To strengthen this manuscript, I would suggest the authors consider adding more visual descriptions of the similarities and differences between the different models. First, by providing a table with the parameter fits for each model per animal. This way, it would be easier to understand what is driving the behavioral similarities between the models and how it ranges between animals. Secondly, while the authors show some simulated behavior (Fig. 2F, G, 3A), not all the models are included, and more analysis would be useful. For example, have the analysis of Fig 1E for all the models, or have a lagged regression of stay/switch based on the past history of rewards (like 3A but as a regression, it might be easier to see more clear differences between models).

We thank the reviewer for their helpful suggestion. The parameters across models may be too different for a simple table comparison, but we have now included standard logistic regression (formulated in Beron et al. 2022), and regression analysis inspired by Vertechi et al. 2020, (Fig. S6)**,** which displayed the "blocking" effect of immediate prior reward at trial t-1 on outcome history effects from more distant trials. We contrasted the regression on mouse behavior with that of the model simulations, and showed that multiple models (including Bayesian models) were able to capture the mouse behavior data qualitatively. In this way, we are able to

summarize the qualitative differences and similarities across models in a salient way, although differences in model parameter spaces make it difficult to compare parameters across the models. In the last paragraph of the first result section, we added:

“More formally, we conducted comprehensive logistic regression analyses to describe how outcome histories influenced behaviors across individual mice, with a particular focus on capturing the decaying impact of historical reward and choice information. By implementing two distinct regression approaches (Beron et al. 2022, Vertechi et al. 2020), we were able to examine how mice integrate recent (1 trial back) and more distant trial history into their decision-making process. The first model explored the general decay of choice outcome weights across trial histories, revealing consistent patterns of how past experiences progressively influence current choices. The second model specifically evaluated whether recent rewards can "block" or reduce the effect of more distant outcome histories. This nuanced analysis allows us to parse the complex cognitive mechanisms underlying mouse behavioral decision-making, providing insight into how mice integrate sequential information to guide their choices. By fitting these models to individual animal data, we can characterize the subtle variations in decision-making strategies across different subjects with higher precision, thus offering a more robust and mechanistic understanding of the experimental results.”

The authors classified all their models into two big families: BRL and RL family (Fig 2A). First, as a minor comment, I would suggest the authors consider a better nomenclature for all their models. With the current acronyms that are similar to each other, it makes it harder to read the manuscript and remember what is being described. Then, I am not sure if I completely agree with their grouping. Although RLCF (the counterfactual RL) does not have a Bayesian inference component, it has been previously described as an approximation of a full Bayesian inference model (Hampton et al. 2007 - https://doi.org/10.1016/j.neuron.2007.07.022). Plus, the counterfactual RL is different from all the other models in the author’s RL family, as it includes some kind of model of the structure of the task.

We thank the reviewer for this point and in response we have modified the figure to better indicate these caveats and connections. However, we would argue counterfactual RL still employs a qualitatively different mechanism compared to Bayesian models. Mathematically, it is still more akin to standard RL models. For example, in a task with closer gap between and high and low value options, counterfactual models fare much differently compared to Bayesian models (Beron et al. 2022). In this revision we opted to continue to work with these three major categories (Bayesian, hybrid, standard and complex RLs) because we think it has helpful explanatory value for the average reader, while imagining that the sophisticated reader will still be able to appreciate the nuances of different models within each category.

Regarding the regressions to predict dopamine response, my understanding from the text is that just the simulated RPEs from the models are included. However, the dopamine response that is being analyzed is also at the same time the reward is being delivered. Therefore, it is necessary to remove the effect of outcome alone (independent of prediction) from the analysis. Can the

authors please show that the same results stand if the current outcome is also included as a regressor?

We thank the reviewer for the question. As a matter of fact, the regression was actually fitted with outcome value as well. We used interaction term between outcome and RPE predictions to represent a separate slope for positive and negative RPEs: `outcome_DA_PT ~ rpe:rpe_pos + rpe:rpe_neg + port_dur:rpe_pos + port_dur:rpe_neg + ego_action + session_num + log__MVMT + log__center_dur`. This regression allows us to test the hypothesis regarding RPE encoding in dopamine, instead of value encoding in dopamine.

In method section, we included: "For model RPE prediction fitting to RPE, we fitted the following regression: `outcome_DA_PT ~ rpe:rpe_pos + rpe:rpe_neg + port_dur:rpe_pos + port_dur:rpe_neg + ego_action + session_num + log__MVMT + log__center_dur`. `rpe:rpe_pos` and `rpe:rpe_neg` encoded positive and negative RPE responses in dopamine, respectively. `ego_action` describes the effect of movement laterality on dLight signal. We included `port_dur, MVMT, center_dur` to movement related components of dLight signals"

It is not clear how R_chosen and R_unchosen are defined. The text mentions "past rewards observed at the selected/opposing port', however, it is not described how many trials in the past, whether there is some exponential decay, etc. Have the authors considered, instead of using the history of past rewards on each port, which has the limitation of the low sample size of R_unchosen as the authors mention, looking at the direction of the RPE based on stay or switch and the outcome on the previous trial (like in Blanco-Pozo et al. 2024 - https://doi.org/10.1038/s41593-023-01542-x), also adding the effect of the current reward as a separate effect as explained before.

We thank the reviewer for the feedback and advice.

We now try to better define R_chosen and R_unchosen. R_chosen was defined as the number of times where the subjects were rewarded in the past 4 trials when at the port that they chose at trial t. R_unchosen reflects the number of times in the past 4 trials the subjects were rewarded when they picked the port opposite from the current selection at trial t.

In the Results section we now state: "Specifically, we modeled the dopamine values, or the predicted RPEs for simulated data, as a linear weighted sum of current reward (Reward), rewards observed at the currently chosen port (trial t) over the past 4 trials (R_chosen) and past rewards observed at the opposite port over the past 4 trials (R_unchosen), conditioned on whether animal switched trials or animal stayed with their prior choice t-1 on the current trial t. On animal switch trials, both BIfp, BRLfwr, and RLCF predicted a positive effect of R_unchosen, but other RL models predicted little to no effect due to a forgetting effect or asymmetrical value updates. LMER fitting revealed a significantly negative effect of R_chosen on dopamine at outcome phase during animal switch trials (slope estimate: -0.128, 95% CI [-0.195, -0.062], p=0.02), and a significantly

positive effect of R_unchosen on dopamine during animal switch trials (slope estimate: 0.126, 95% CI [0.035, 0.217], p=0.03, Fig. 4I).”

The alternate analysis proposed by the reviewer is difficult in practice. Due to the 75-0 paradigm, we have a limited number of trials where mice switch to a new port and get rewarded after observing rewards in the previously chosen port (reward-switch-reward sequence) (This is quantified in the text from “discussion” below). Therefore, we instead still use regression in the section on dopamine signals to highlight the qualitative difference between Bayesian inference based strategy and other RL strategies.

“In this design, in any given block, an inferior port always yields no reward. Consequently, mice rarely switch to a new port and get rewarded after observing rewards in the previously chosen port. Previous work has noted that inference-based cognitive models are capable of decreasing the expectations about alternative options after observing a reward in selected options. Therefore, mice using inference would exhibit a higher RPE following a reward in a new port after also observing a reward in the old port, a reward-switch-reward sequence. Yet in our dataset, there are only 94 trials initiating such sequences out of 30,707 trials in total, across multiple animal sessions, giving us limited power to address this prediction.”

In the methods section, it is mentioned that some trials were dropped. Please can the authors add what is the proportion of these? For the photometry analysis, it seems that not all the data was included, can the authors also specify how many sessions were excluded? And from the included, how many trials were dropped?

We thank the reviewer for the careful feedback and apologize for not being clear. We only recorded neural data every other day or every third day to prevent signal bleaching. We noted this in Methods section:

“During the learning switch phase, we start plugging in fiber implants without recording to get mice accustomed to moving and behaving with fibers. Starting from the first “full task" session, we alternate between a left hemisphere recording, right hemisphere recording, and no recording schedule. The no recording day was implemented to avoid signal bleaching. ”

We also filtered out trials that showed long durations, possibly due to grooming or other off task behavior, by removing trials with gaps longer than 1s between two nose pokes at side ports, or with intervals longer than 6s between outcome and next trial initiation.
We rewrote the methods on these issues to improve clarity.

“To minimize the effects of intertrial events on neural signals, we selected sessions with center_dur <= 0.8 (mice lingering less than 0.8 seconds at center port), port_dur <= 6 (intervals shorter than 6s between outcome and side port departure), MVMT <= 3 (intervals shorter than 3s between final side port departure and next trial initiation). We also filtered out trials with SO_lat01 > 1, where the trials had gaps longer than 6s between two nose poke bouts at side ports. These trials typically consist of trials where mice revisited the port multiple times, or simply mistriggered the infrared sensors used to measure nose pokes, biasing the task

structure. After this cleaning, we were left with 20,952 trials (~68% of the dataset) across 34 sessions with valid dopamine recordings."

Have the authors considered adding the asymmetric impact of rewards and unrewards into their Bayesian models? For example, in their BRLfwr, have they tried to include asymmetric learning rates?

We thank the reviewer for the thoughtful suggestion. We intentionally did not include asymmetric learning rate as a mechanism in BRL so that we can highlight the effectiveness of Bayesian mechanism "alone" to explain mice behavior and neural data. In this way, we are able to compare Bayesian inference against other mechanisms alone.

In a further revision, we could explore this option as well, but feel it is out of scope at present.

Can the authors please explain why they baseline-corrected their z-scored dLight signals? Also, why subtract the signal at outcome time, which is the time when a lot of information about the state of the task is being provided?

We thank the reviewer for the careful comment. Subtraction by signal outcome uniformly across all trials aimed to isolate the changes in dopamine levels due to outcome alone, removing pre-outcome information that may interfere.

Since our sampling interval is 50ms and there are often delays due to multisynaptic connections and sensor kinetics, this approximation is appropriate. For dLight signal processing: we borrowed methods from Martinova et al. 2019, which is also qualitatively similar to methods discussed in Simpson et al. 2024. In the method section, we described the preprocessing steps: "we first subtracted the channel specific trends by approximating a smooth fit using airPLS algorithm across both channels. Then we perform a robust linear regression from reference channel to signal channel, and subtract the fitted 415nm artifact signal from 470nm channel recordings and obtain the processed dLight signals. All dLight signals are subsequently z-scored to account for extraneous variations between sessions."

Minor comments:
line 307: I would not say VTA, as there is a full debate into the relation between VTA dopamine activity and axonal activity and concentration in the striatum. Maybe change for NAc core dopamine concentrations.

Thanks, we have modified the manuscript accordingly.

line 692: 'Probswitch phase' —> I guess that refers to the full final task?

Thanks for the clarification and yes, we modified the manuscript and refer to it as "full task" to clear any confusion.
