# Supplementary material for: Nucleus accumbens dopamine release reflects Bayesian inference during instrumental learning
Source: PLoS Comput Biol. 2025 Jul 2;21(7):e1013226. doi: 10.1371/journal.pcbi.1013226 (PMC12233953; doi:10.1371/journal.pcbi.1013226)
Supplement: Attachment — Submitted filename: response_to_reviewers.pdf [file pcbi.1013226.s010.pdf]

Reviewer #1: I appreciate the efforts the authors have put in, and they addressed most of my concerns. The paper should be ready for publication once the following question is addressed:

Could you clarify if Fig. S7 is fitting dopamine responses or behavior? I assume it is dopamine, but this should be explicitly stated in both the figure legend and the manuscript. While I understand that the learning process is not the focus of this study, it would still be valuable to fit behavioral data from the pretraining period, even though dopamine data were not collected during that time.

We thank the reviewer for their kind comments and insightful feedback. Fig S7 shows the model fit to behavioral data across multiple training sessions in the "Full task (75-0)" phase. We modified the supplementary figure title to "Model fitness results for behavioral data across sessions" to highlight this. We also included the description in the Discussion section of the main text:
"**Mice started with blocks with 100% reward probability, which was brought down in stages to 90%, 80% and then 78%, before the "full task" at 75%. Since this pre-training took approximately 10 days on average, it may have been enough for mice to develop Bayesian inference strategies before dopamine recordings started. When we fit models to single session behavioral data during the "full task" phase, we did not observe any changes consistent with transitions from one cognitive strategy to another across days in the full task (Fig. S7). As a result, our current dataset cannot address the question of whether, in the earliest days of pretraining, mice transitioned from initial reinforcement learning to a more sophisticated belief-state inference approach.**"
We also agree with the reviewer's suggestion in analyzing the model fitness to behavioral data from pre-training period – it would indeed be quite informative if we could see "emergence" of belief state behaviors in this process. We are planning to address this question in an upcoming study with a larger scale behavioral study across a larger cohort of animals.

Reviewer #2: This is a revised version of a previously submitted manuscript, in which the authors analyze a wide range of models, Bayesian and more complex RL models, against the simplest RL model to explain mice behavior and dopamine levels in the Nucleus Accumbens core in a 2ABT. This revised version's main changes are in the introduction, which places this work within the context of previous literature and clearly states the aims of the work. The authors have also added another model and extra analysis in supplementary material, which I believe helps in the comparison between models and data.

I would like to thank the authors for their replies and for taking into account my comments. I agree with the authors that the wide range of models and replication is useful and important for

the community and is within the scope of PLOS Computational biology. Although their results are consistent with previous literature, showing that Bayesian models can explain behavior and dopamine signals, my main critique was that this work, despite using a wide range of models, is not able to conclusively distinguish between Bayesian and more complex RL, even if Bayesian models seems to better (but not completely) explain dopaminergic RPEs than the other RL models. I think it further highlights the complexity of modeling mice behavior and dopamine signaling using these models, and that probably, a combination of these models (or even new models) might be necessary to fully capture behavior and dopamine.

We thank the reviewer for their thoughtful comment. While we agree that behaviorally we cannot completely distinguish Bayesian models and complex RL models, we believe that this result precisely highlights the importance of model fitness validation using both behavioral and neural data. While two classes of models, as well as the hybrid model can explain similar amounts of behavioral variances, the computation in specific neurons may differ. The increase in explanatory power in dopamine data by Bayesian and hybrid model predictions strengthens the argument that Bayesian computations are likely leveraged by neural circuits for value estimation and error signaling.

To ensure we reflect the reviewers' point on modeling complexity, we included these sentence in the last paragraph before conclusion:

"We believe that better discrimination between these models can come from theory-guided experimental design, possibly using a more complex task that places a greater demand on learning policies within each belief state \cite{akam_simple_2015, miller_dorsal_2017}. Moreover, in future work, more careful examinations of distinct cognitive mechanisms may also allow us to develop new models to further illuminate the questions raised in our study."

That's why I think the claims of the manuscript, especially at the beginning of the discussion, do not precisely align with their results, and I think it could be further revised to highlight the complexity and difficulty in fully capturing behavior and dopamine using these models. For example, line 446 states that Bayesian models outperformed RL models in explaining mouse behavior. However, I don't think that is completely clear in the results (e.g. in the new analysis in S6, one could say that RLFQ3p actually captures better animals' choice behavior). This further shows that behavior and dopamine signaling do not necessarily need to be aligned, as different systems might be differently involved in producing behavior as animals potentially shift from goal-directed to more habitual control.

We thank the reviewer for their helpful suggestions, and apologize for the lack of clarity in our wording. We want to highlight that behavioral data showed that models that incorporate Bayesian inference for state representations fit better to behavioral data than standard RL models, not complex ones; but the dopamine data fitting allowed us to further arbitrate between the models. We modified the discussion section to fully reflect this:
"Both Bayesian models (the pure Bayesian inference model and the belief state RL model) were able to capture this balance and outperformed standard RL models in explaining mouse behavior (Fig. 2C, Fig. 3C). While behavioral data alone was not

sufficient for us to arbitrate among Bayesian models and complex RL models, Bayesian models were able to provide a more accurate account of the dopamine release events in the NAc core following choice outcomes (Fig. 4G-J).

As to the last point, we agree that different systems may be driving behavior at different times. We are happy to reflect this in our manuscript discussion. However, we do not think transition to habits is a good fit in this specific case. While it is true that goal directed behavior in response to specific stimuli in a stable environment can become insensitive to outcome delivery with long term training (definition of a habit), our task design provides no informative cues and requires regular flexible updating, so it cannot be solved by habitual behavior in its most basic definition (if switching can become a habit is another level of discussion). Our data counter this idea too by showing that mice maintained their behavioral adaptiveness in later sessions. Finally, we can positively address the more general idea of alternate strategies emerging with long periods of training in Fig. S7 which shows performance cannot be explained as animals transitioning to a WSLS strategy or a greedy RL process.

Consequently, in response to the reviewer's comments, we do not go into habit formation explicitly, but instead we try to acknowledge the possibility of later changes in strategy in a rewritten S7 legend "To test if there was meta-learning across sessions, we fitted cognitive models to mouse behavior across multiple sessions. We did not find evidence of any significant and consistent increase in model fitness across multiple sessions. We speculate that mice were able to learn the inference structure of the task during a brief pre-training phase, when the reward probability of the high value option changed from 90%, 80%, to 78% (Fig. 1B). Model fitness of Bayesian models did show some decline in some later sessions but then recovered, suggesting that there was no sustained change in behavioral strategy after extended training."

We thank the reviewers for their time and hope these changes make the paper more thorough.

As for some other minor comments, I think it would be good to also show RFLR in S6, as the authors state that it is similar to RLFQ3p. I think it would be useful, the same way it is useful to see in this plot, that both Bayesian and RLCF models show qualitatively similar simulated behavior. Finally, I may have missed it, but supplementary figures S3-6 do not seem to be referenced in the main text

We thank the reviewers for their helpful comments. We did not initially include RFLR results due to their mathematical similarity to RLFQ3p. Now we included RFLR as a model and removed BIfp model, due to its similarity to BRLfwr. Moreover, we included Figures S3-6 references for consistency and sincerely appreciate your thoroughness.
